# High-Frequency Acoustic Imaging Using Adhesive-Free Polymer Transducer

**DOI:** 10.3390/polym13091462

**Published:** 2021-04-30

**Authors:** Abhishek Ranjan, Chengxiang Peng, Sanat Wagle, Frank Melandsø, Anowarul Habib

**Affiliations:** 1 Department of Physics and Technology, UiT The Arctic University of Norway, 9019 Tromsø, Norway; abhishek.ranjan@uit.no (A.R.); chengxiang_peng@sina.com (C.P.); frank.melandso@uit.no (F.M.); 2 Elop AS, Nordvikvegen 50, 2316 Hamar, Norway; Sanat@elop.no

**Keywords:** cartilage, acoustic microscopy, ultrasound, P(VDF-TrFE), transducers

## Abstract

The piezoelectric polymer PVDF and its copolymers have a long history as transducer materials for medical and biological applications. An efficient use of these polymers can potentially both lower the production cost and offer an environment-friendly alternative for medical transducers which today is dominated by piezoelectric ceramics containing lead. The main goal of the current work has been to compare the image quality of a low-cost in-house transducers made from the copolymer P(VDF-TrFE) to a commercial PVDF transducer. Several test objects were explored with the transducers used in a scanning acoustic microscope, including a human articular cartilage sample, a coin surface, and an etched metal film with fine line structures. To evaluate the image quality, C- and B-scan images were obtained from the recorded time series, and compared in terms of resolution, SNR, point-spread function, and depth imaging capability. The investigation is believed to provide useful information about both the strengths and limitations of low-cost polymer transducers.

## 1. Introduction

Scanning acoustic microscopy (SAM) is a widefield non-destructive and non-invasive technique that has been widely used over several decades for surface and subsurface microscopic imaging especially for industrial and biological specimens [1,2,3]. SAM has the potential to employ high-frequency waves which are transmitted via a coupling medium into the sample and become reflected based on the stiffness of the sample [4]. SAM can for instance be used to observe internal structures, subsurface features, structural characterization of materials, and detect changes in the elastic properties of solids [5,6].

The typical commercial transducers used in SAM are made up of ceramic, single crystals, or thin films of piezoelectric materials. Polyvinylidene difluoride (PVDF) and its copolymer with trifluoroethylene P(VDF-TrFE) are ferromagnetic materials that inherently possesses several benefits for an acoustic transducer. These are flexible materials which allow a high degree of physical focusing without lenses [7]. Polymer transducers typically offer wide bandwidth or short impulse response [8], and much a better acoustic impedance match to biological tissue than ceramic-based transducers. The copolymer P(VDF-TrFE) is commercially available in different mass mixing ratios where a ratio around 70 to 30 between PVDF and TrFE is known to produce the highest piezoelectric activity [9]. Ceramic-based transducers, on the other hand, normally have advantages in terms of a higher dielectric permittivity, higher electromechanical coupling, and lower loss factors.

Most commercial high-frequency transducers are produced by depositing a thin layer of the piezoelectric material on the flat side of the buffer rod. A concave spherical sapphire lens rod is typically used to focus acoustic energy through a coupling medium (i.e., water) onto the sample plane. The pronounced impedance mismatch leads to reduced sound transmissivity, significant bandwidth reduction, and geometrical aberration of the focusing beam [5]. For PVDF and P(VDF-TrFE), on the other hand, focusing can be archived by curving or pressing these flexible materials under production. The close match between the polymers and water impedances and a relative high material loss typically yields significantly higher bandwidths than for comparable ceramics and ceramic composites. The mismatch in acoustic impedance for ceramic-based transducers also requires one or more matching layers, which again increases the complexity and production cost.

To investigate the transducers’ capabilities for medical imaging, a test sample of articular cartilage was chosen. Articular cartilage is a type of connective tissue that serves as a cushion between joints. It is a hyaline, helping bones to glide over each other with low friction [10,11]. Blood vessels, lymphatics, and nerves are also absent [12]. The indication of degeneration of cartilage can be seen clearly from the changes in biomechanical properties e.g., in rheumatoid arthritis or osteoarthritis (OA) [13,14]. OA is a degenerative joint disease in which the articular cartilage gradually degrades. The prevalence of OA can be viewed as an age-related phenomenon, as chondrocyte function changes with age. The symptoms include pain, stiffness of the joint, and weakness in muscles. Several morphological and structural modifications occur during the development of OA [15,16,17,18].

Only severe cartilage lesions, which are a feature of later stages of OA, can be detected using clinical non-invasive imaging modalities such as radiography, magnetic resonance imaging, computed tomography, and conventional echography [19,20,21,22]. Since superficial cartilage degradation is the first sign of OA, its early detection would be of crucial diagnostic importance. Changes in the cartilage’s acoustic properties have been proposed as sensitive indicators of degeneration.

In comparison to other imaging modalities, acoustic methods can probe both surface and subsurface elastic properties of biological samples, such as chondrocytes in cartilage, which tells us about the status of cartilage [23,24,25,26,27]. The majority of previous articular cartilage acoustic imaging studies were conducted on thin samples using commercial high-frequency acoustic transducers [28,29]. We used a layer-by-layer deposition method to create an adhesive-free transducer made of P (VDF-TrFe) material with a smaller aperture diameter and lower f-number. The main goal of this study was to compare the performance of commercial and in-house transducers using high-frequency ultrasonic imaging of much thicker cartilage specimens (mm thick).

## 2. Materials and Methods

**Figure 1:**
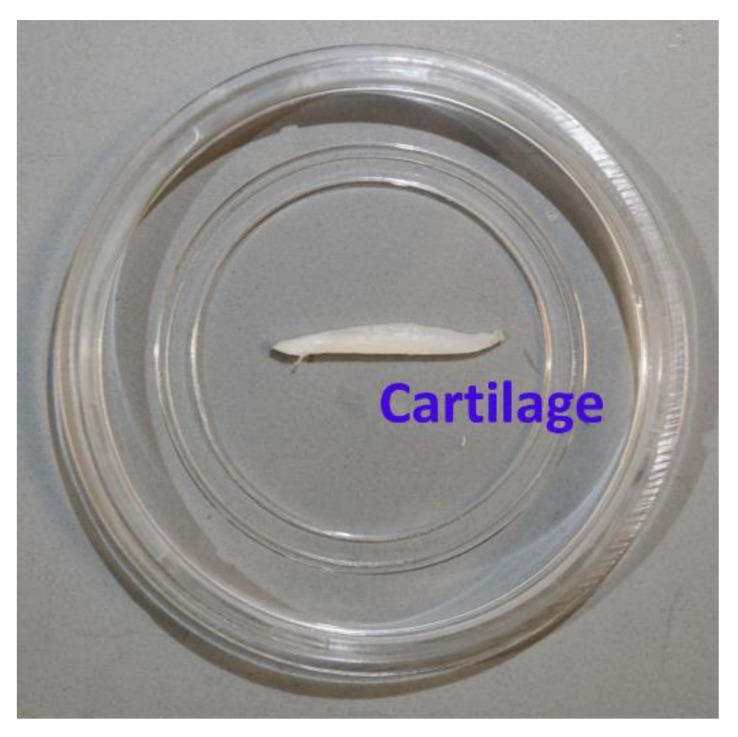
Articular cartilage sample used in the experiments for acoustic imaging for both the in-house fabricated and the commercial PVDF transducer.

A non-degenerated human cartilage sample with a smooth and unblemished surface was obtained from the University Hospital of Northern Norway, Tromsø, Norway (REK Nord 2014/920). The sample came from the patients (age between 50 to 60) who underwent a complete knee replacement. The patients with inflammatory joint diseases and advanced osteoarthritis were excluded from the study. The Regional Ethical Committee of Northern Norway (REK Nord 2014/920) approved the study. Written consent was provided from all patients for the use of their cartilage for further research. We prepared the sample by slicing them carefully, which resulted in a sample of around 1.25-mm thickness and then stored them in the PBS (phosphate buffered saline) solution. The cartilage sample was placed inside a Petri dish (ibidis-50 mm) made up of polymer. The sample was fixed on the surface of the Petri dish using sticky tape. The Petri dish with cartilage was filled with water to improve acoustic coupling since air is a poor transmitter of acoustic energy coming from transducers. Figure 1 represents the optical image of a cartilage sample.

## 3. Transducer Fabrication

**Figure 2:**
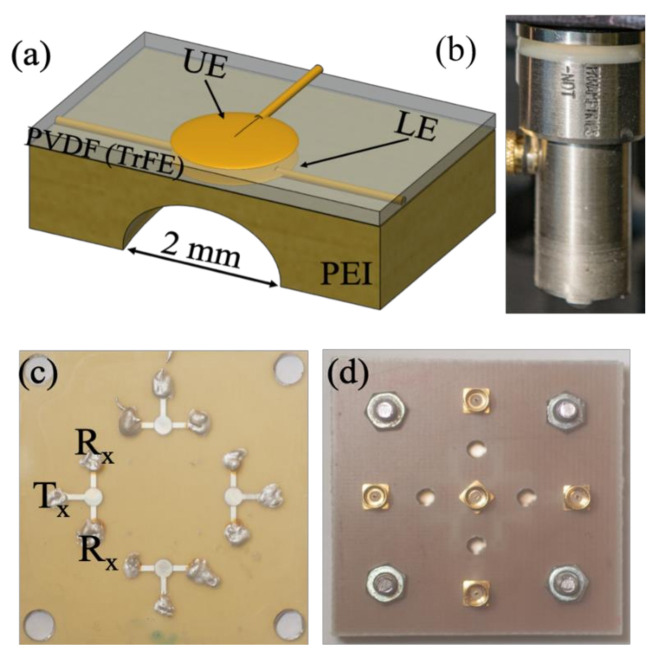
(**a**) Schematic representation of the fabricated in-house P(VDF-TrFE) copolymer transducer, and (**b**) image of the commercial Olympus PVDF transducer, both with center frequencies around 40 MHz (**c**) Optical image of a transducer panel containing four transducers, and (**d**) connection RF plugs for the transducers mounted on a printed circuit board for further cable connection to the signal generator and receiver.

The fabrication process of the P(VDF-TrFE) transducer began with an engraving of polymer polyethyleneimines (PEI) substrate with dimensions of 30 × 30 mm^2^ and 0.85 mm thickness with four spherical cavities of 2 mm diameter. After the milling process, the spherical cavities were cleaned with ethanol to remove unwanted material and grease. In order to increase the wettability of the substrate, a plasma cleaner was employed. Sputtering was done through a high-resolution metal mask to get the lower electrode (LE) layer. The top of the substrate was pre-treated and sputtered through a metal shadow mask to yield an 80-nm thick patterned-Au layer acting as the lower electrode. Subsequently, spin-coating was done on top of the patterned electrode fluid phase P(VDF-TrFE) (77:23, molar ratio) dissolved in a suitable amount of solvent. Later on, the spin-coated P(VDF-TrFE) was degassed under vacuum and thereafter annealed at 130 °C. Finally, an upper electrode was sputtered on the top of P(VDF-TrFE) by repeating the process used for the lower electrode. The sputtered metal layer was then verified using surface nano- profiler. Figure 2 presents the schematic representation of our in-house fabricated P(VDF-TrFe) transducer [30].

**Figure 3:**
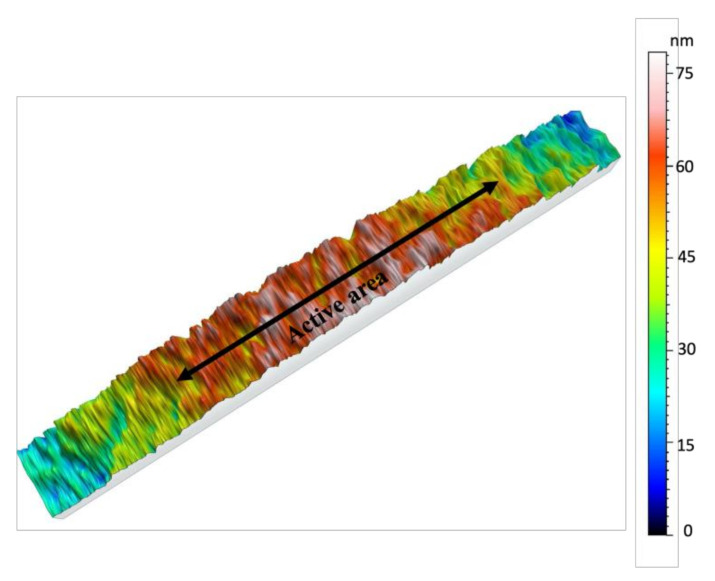
Surface roughness of the metalized electrode and P(VDF-TrFE) area of the fabricated transducer. Scan area- 90 × 600 μm^2^. Active area indicated in the figure refer to the metalized electrode area.

Figure 3 demonstrates the surface nano profiling of the metal electrode. The scan area was 90 × 600 μm^2^ of the metal electrode area and on the spin-coated polymer area. Some height variations were visible outside (polymer area) of the active metalized area. These height variations occurred due to diffusion during sputtering with shadow mask. This is a very common problem sputtering with a metal shadow mask.

Later on, the upper electrode (UE) was deposited silver on the top of P(VDF-TrFE) with the patterned metal mask. The thickness of the upper and lower electrode was measured to be 80 nm, whereas the thickness of the anneal PVDF copolymer film thickness was estimated to be 12 μm using a KLM nanoprofiler. Each of the transducers in the substrate was connected directly onto a PCB using small spring contacts for poling and characterizing the acoustic response. For the poling process and characterizing the acoustic response of the fabricated transducer, each of the transducers in the substrate was connected directly onto a printed circuit board (PCB). Small spring contacts were employed in order to minimize additional inductive and capacity effects caused by open connectors. For making the P(VDF-TrFE) layers piezoelectric, they were polarized at room temperature by connecting a high voltage AC source to the lower electrodes, while the upper ones were grounded [31,32].

## 4. Experimental Setup

**Figure 4:**
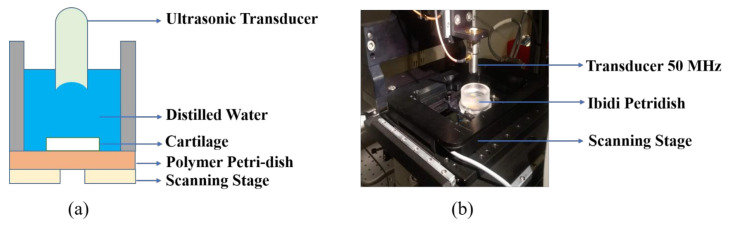
(**a**) Schematic diagram of our setup with ultrasonic transducer kept above the cartilage in Petri dish. (**b**) Camera view of our setup showing transducer, Petri dish, and scanning stage.

The experiments were performed on an inverted microscope (Leica Dmi8, Wetzlar, Germany) assembled with a custom-designed high precision scanning platform (ASI MS-2000, Eugene, OR, USA) and other components capable of producing B-scan and C-scan images. A detailed overview of the experimental setup can be found in our previous paper [32]. The ultrasonic functionality in scanning acoustic microscope was given using FlexRIO modules and field programmable gate arrays (FPGA) hardware from National Instruments (Austin, TX, USA). This hardware constitutes an arbitrary waveform generator (AT-1212), a 3W RF-amplifier (E&I 403LA) for pulse excitation, and a high-speed (1.6GS/s) 12-bit digitizer for pulse recording (NI-5772). All of the scanning operations including transducer movements and stage translations were controlled using the LabVIEW (2016) program. The cartilage specimen inside the Petri dish was kept on the scanning platform; the ultrasonic transducer fixed above the stage was focused onto the sample and the stage was scanned in raster mode. We used commercial and in-house fabricated transducers and the comparison based on different parameters is shown in Table 1 below. Figure 4 represents the schematic view and the camera view of our experimental setup.

**Figure 5:**
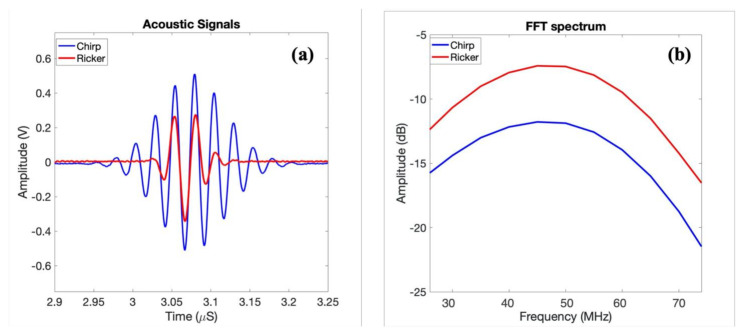
(**a**) Ultrasonic measurements of the focused transducers reflections from the glass plate at a focal point of the in-house transducer; (**b**) frequency spectra in dB scale corresponding to (**a**).

In order to explore the advantages of using coded waveforms for biological samples, both a Ricker wavelet (second derivative of a Gaussian signal) and a long chirp-coded waveform were implemented on the imaging system. Figure 5 shows the ultrasonic measurements of the focused transducer showing reflections from the glass plate at the focal point of in-house transducer for chirp and Ricker wave with its frequency spectrum. Using the long-coded sequences, e.g., chirp waves, can increase the signal-to-noise ratio (SNR). The increased SNR can make it possible to image deeper and at the same time obtain good resolution. These waveforms were also investigated using both an in-house fabricated P(VDF-TrFE) transducer and a commercial PVDF transducer, both with center frequencies around 40 MHz.

The average central frequency of the in-house transducer was measured to be (48.5 ± 1) MHz, with lower and upper −6 dB frequencies of approximately 25 and 76.5 MHz, yielding a bandwidth of 94.2%. This was measured from acoustic pulse measurements as shown in a previous paper where the acoustic reflection of the focused transducers from the glass plate at a focal point was shown. The frequency response has been estimated as the ratio between the output and input power from the glass reflector.

The focal length of the fabricated P(VDF-TrFE) transducer was measured using a hydrophone system. The focal point of the polymer transducer was found to be located approximately at 2.5 mm from the cavity of the transducer. In order to confirm the measurement of the focal point, a simulation was performed using COMSOL (5.2) multiphysics software in our previous paper [33]. The experimental setup is given as below.

**Figure 6:**
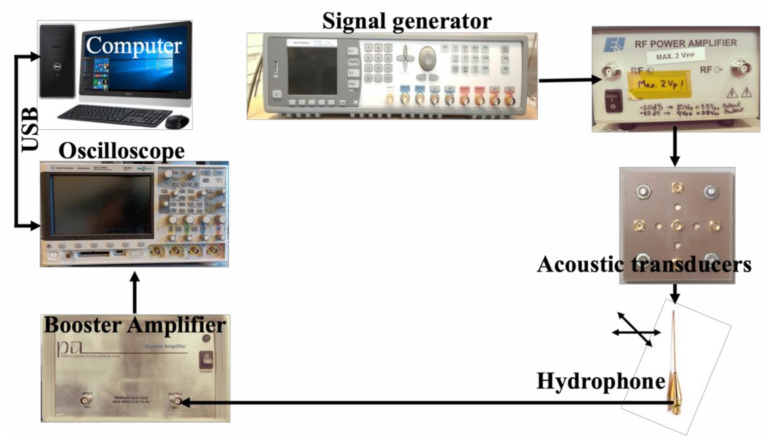
An experimental setup for the hydrophone system for the focal length measurement.

An overview of the individual components used in the focal length measurement is demonstrated in Figure 6. These components consisted of the signal excitation side, an arbitrary function generator (Tektronix AFG 3102) used to generate the required pulse form (chirp and Ricker), and a radio frequency (RF) amplifier (Electronics & Innovation: 403LA (Rochester, NY, USA) employed for magnifying the pulse-form before delivery into the transducer. On the receiver side, the hydrophone system included a 75-μm needle hydrophone (precision acoustics) with (1-30) MHz bandwidth with an internal amplifier, an additional booster amplifier, and an oscilloscope (Agilent 3024A, Santa Clara, CA, USA). This oscilloscope performed averaging 256 pulse shootings, digitizing the signal, and storing it into a PC via a universal serial bus (USB) connection.

## 5. Results and Discussions

A prepared slice of articular cartilage (Figure 1) was used as a test sample to explore differences between the investigated transducers. For each transducer, both Ricker and chirp-coded pulses were used as an excitation pulse, and the recorded time series from these pulses were averaged 16 times to obtain noise reduction. The average central frequency of the transducer was measured to be (48.5 ± 1) MHz, with lower and upper −6 dB frequencies of approximately 25 and 76.5 MHz, yielding a bandwidth of 94.2% [33]. 

The commercial transducer peak frequency was around 40 MHz and the frequency bandwidth lay between 24.5 and 51.87 MHz as given on the Olympus website [30]. The lateral resolution in scanning acoustic microscopy generally depends on the acoustic wavelength and the f-number of the transducer [2]. Lateral resolution is defined as:

(1) Lateral resolution=f−number×acoustic wavelength

We compared the lateral resolution for both the transducers, which was 60 μm for the commercial transducer and 50.25 μm for the homemade transducer theoretically. We also compared the depth of focus for both the transducers which could be defined as products of acoustic wavelength and square of the f-number [34]. The −6 dB for example, can be estimated from:

(2) $$\mathrm{Depth}\;\mathrm{of}\;\mathrm{focus}\;(\mathrm{DOF})=9.68\times \mathrm{acoustic}\;\mathrm{wavelength}\times {\left(f-\mathrm{number}\right)}^{2}$$

The depth of focus was calculated to be 1.5 mm for the commercial transducer and 0.90 mm for the in-house transducer. The averaged backscattered signals were digitized into time series containing 2048 samples for each pixel in a xy-scan matrix and saved to an HDF5 file format, for further processing in MATLAB. This processing includes, for example, wave compression of the coded waveforms, which was done using a Wiener filter in the Fourier domain, as previously described in our paper [17,32]. After wave compression, the time series were finally processed into C-scan and B-scan images. Examples of C-scans taken at a shallow depth into the cartilage sample are shown demonstrating the scans for the commercial and in-house transducers, respectively, driven by the Ricker pulse. 

**Figure 7:**
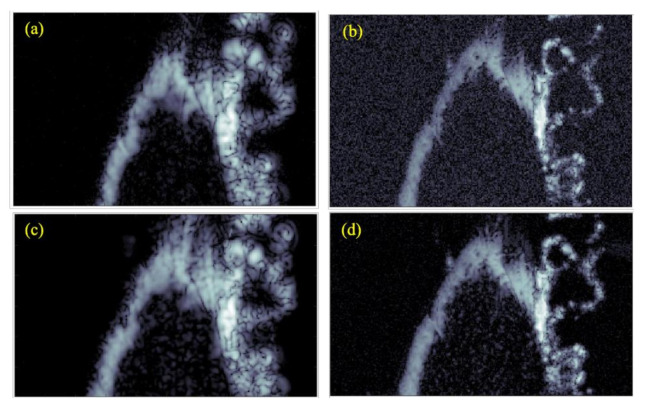
C-scan images generated of the test sample using different transducers and excitation pulses. These are (**a**) commercial transducer with Ricker wavelet, (**b**) in-house transducer with Ricker wavelet, (**c**) commercial transducer with chirp code, and (**d**) in-house transducer with chirp code. All images have a dimension of 3 × 5 mm^2^.

**Figure 8:**
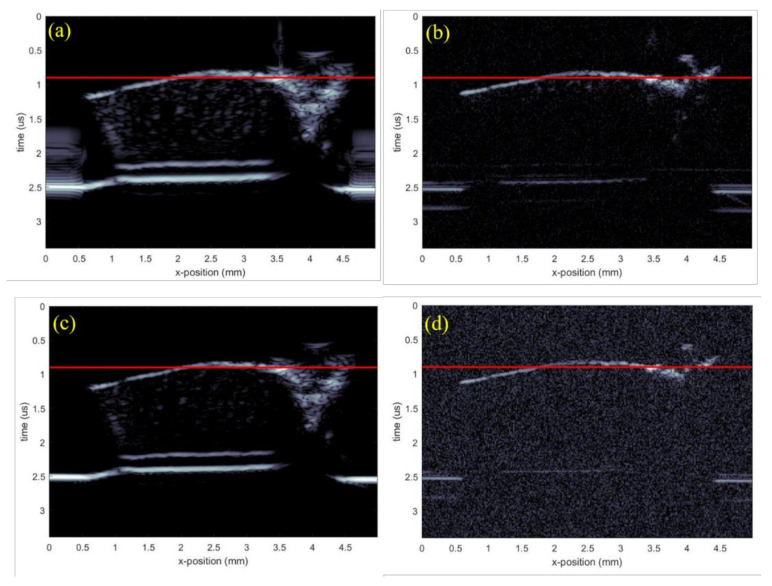
B-scan images generated of the test sample using different transducers and excitation pulses. (**a**) Commercial transducer with Ricker wavelet, (**b**) in-house transducer with Ricker wavelet, (**c**) commercial transducer with chirp code, and (**d**) in-house transducer with chirp code.

The corresponding results when using a chirp excitation pulse are shown in Figure 7c,d. B-scans of the test sample are shown in Figure 8a,d, with the figures corresponding to the transducer-waveform combinations previously used in Figure 4. The red lines in Figure 8 show the spatial locations where the C-scans in Figure 7 were taken. This shows that the backscattered signals were generated from the internal structures, i.e., arising from the cells of cartilage. From Figure 7 and Figure 8, we observe by comparison, that the commercial transducer yielded a significantly higher SNR than the in-house transducer. Single-pixel (salt and pepper) noise is, for example, clearly visible in all domains of Figure 7b, while it cannot be spotted in Figure 7a. The observed difference in SNR is probably strongly related to the difference in transducer aperture sizes listed in Table 1. The signal-to-noise ratio was calculated from the ratio of the average value of the signal to the standard deviation of the signal. The SNR was calculated for all the cases and the results are shown below in Table 2.

We can also observe that there are some differences in the physical properties between the polymers and the ceramic based on the comparison of electromechanical coefficients [35]. PVDF has the advantage over other types of transducer in terms of acoustic impedance, high mechanical flexibility, and modest piezoelectric properties, making it suitable for high-frequency imaging. The pronounced impedance mismatch leads to reduced sound transmissivity, significant bandwidth reduction, and geometrical aberration of the focusing beam. Most of the commercial polymer transducers employed a non-conducting epoxy as a baking material for fabricating the transducers. The additional epoxy layers may have contributed to an increased inhomogeneity (e.g., through-thickness variation) and high surface roughness. The homemade transducer presented here followed a new approach of fabricating high-frequency P(VDF-TrFE) focusing transducer by employing adhesive-free layer-by-layer deposition technique.

The commercial transducer also explored more details from the inner parts of the cartilage, especially from the deepest parts [36]. This is clearly seen, e.g., by comparing the results for the chirp waves shown in Figure 7c,d. Here, Figure 7c shows elliptical-shaped scatters originating from cells through the entire sample, while these scatters were visible only towards the upper surface in Figure 7d.

On the other hand, the elliptical scatters from the in-house transducer appeared significantly smaller than from the commercial transducer, suggesting that the point-spread function from the latter was larger. However, the penetration depth remained the same because both the transducers were operating at the same frequency, evident from the B-scan in Figure 8. This came from the complex inverse relationship between spatial resolution and penetration depth. It was also possible to observe finer details on the C-scan from the in-house transducer, which, together with a smaller point-spread function, suggest that this transducer had a significantly better resolution. This result can to some extent be explained by the smaller f-number of the in-house transducer.

**Figure 9:**
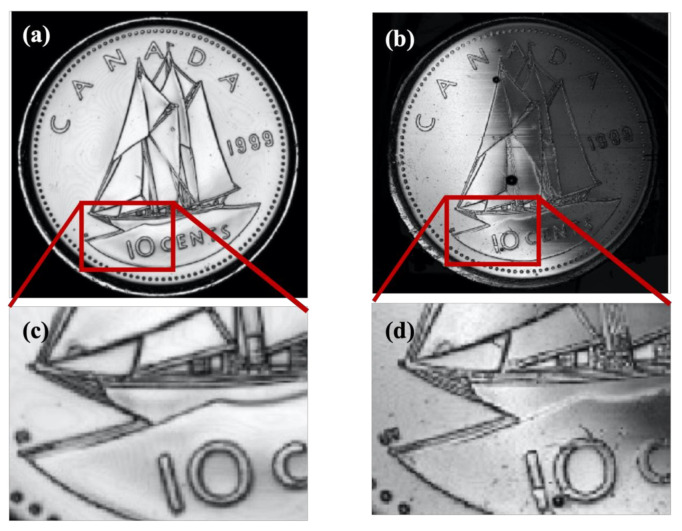
(**a**) C-scan of Canadian 10-cents coin by commercial polymer transducer; (**b**) C-scan of transducer by in-house polymer transducer. Images have a dimension of 20 × 20 mm^2^. (**c**,**d**) are the magnified views of (**a**,**b**), respectively.

**Figure 10:**
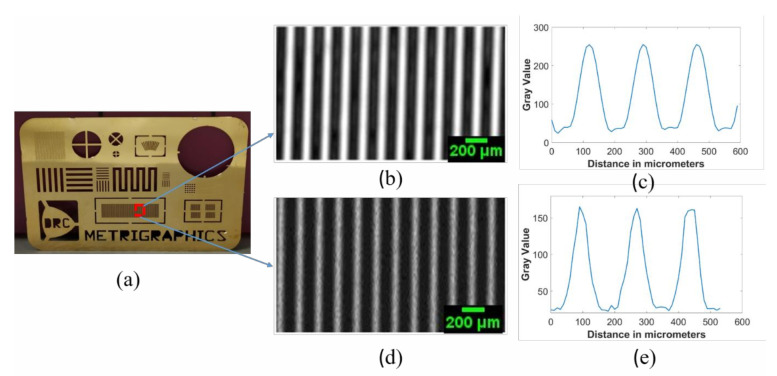
(**a**) Optical image of the resolution chart; (**b**) C-scan image from commercial transducer; (**c**) profile plot of the image taken from commercial transducer; (**d**) C-scan from in-house transducer; (**e**) profile plot of the image taken by in-house transducer. Scale bars with length 200 μm are given in images (**b**,**d**) where each pixel has a 10-μm physical size.

We also imaged two other test samples with both transducers in order to confirm the conclusions deduced from the cartilage image. These samples included a Canadian 10-cent coin, as shown in Figure 9, and metal film, as shown in Figure 10. The metal film, referred to as the resolution chart, was patterned with high-resolution etching by Metrigraphics (now Cirtec Medical). The in-house transducer detected finer details, as evident from the picture shown below in Figure 9. We also magnified the image on a particular region where some finer details were present. For instance, the rope structure was clearly visible in Figure 9b (home-transducer image), which was not visible in the image taken from the commercial transducer in Figure 9a.

By comparing the images in Figure 9a,b, we noticed fine details on the coin surface for the in-house transducer, which was not clearly visible for the commercial one, suggesting a better resolution for the in-house transducer. The in-house transducer also yielded a systematic amplitude reduction over the coin from left to right, which was believed to be caused by a slight tilt between the coin surface and scanning plane. We also noticed that the very thin stripes selected inside the square shown in Figure 10a appeared narrower in the image taken from the in-house transducer than the commercial transducer. This confirms an improved resolution for the in-house transducer. However, the image from the commercial transducer had a much higher signal-to-noise ratio which is believed to be mainly caused by the much larger aperture area. Figure 9 and Figure 10 further confirm our qualitative and quantitative comparison of image quality based on resolution, signal-to-noise ratio, depth of focus, and point-spread function.

## 6. Conclusions

We produced a small aperture P(VDF-TrFE) copolymer transducer appropriate for high-frequency imaging which was compared to a commercial PVDF transducer. To investigate the differences between these transducers, the transducers were used to image a medical sample (articular cartilage) and two other mechanical test objects (coin and etched film). The results demonstrated that the low-cost homemade P(VDF-TrFE) transducer is capable of producing images with a resolution for the cartilage test sample that was superior to the commercial transducer, e.g., with visible cell structures in the upper part of the sample. This result was also confirmed from the coin and resolution chart images, reinforcing much finer details with the in-house transducer. The improved resolution of the in-house transducer was also confirmed with a smaller point-spread function. However, the drawback with the in-house transducer was the much smaller SNR, which inhibited penetration deep into the cartilage sample. The in-house transducer was therefore not suitable for volume imaging of thick samples. The B- and C-scan images taken with both transducers clearly showed that the chirp-coded waveform was superior to the Ricker wavelet in terms of SNR and dynamical range. It was also believed that the SNR for the in-house transducer could be improved significantly by several means. This includes, for example, redesigning the systems’ pre-amplifier for the higher electrical impedance and increasing the amplitude of the driver pulses. Another option that would compensate for the in-house transducer’s much lower aperture area would be to use a multi-layer transducer where both improved shielding and more layers would increase the SNR. 

## Figures and Tables

**Table 1:** In-house fabricated P(VDF-TrFE) copolymer and commercial PVDF transducers’ specification.

Transducer	Commercial PVDF	Fabricated P(VDF-TrFE)
Focal distance	12.7 mm	2.6 mm
Aperture diameter	6.35 mm	1.6 mm
f-number	2	1.625

**Table 2:** SNR comparison in all transducer cases.

Commercial Transducer (Chirp)	Commercial Transducer (Ricker)	Homemade Transducer (Chirp)	Homemade Transducer (Ricker)
22.6	14	9.31	4.86

## Data Availability

The data presented in this study are available on request from the corresponding author.

## References

[1] Korpel A., Kessler L.W., Palermo P.R., Korpel L.W.K.A. (1971). Acoustic Microscope operating at 100 MHz. Nat. Cell Biol..

[2] Briggs A., Kolosov O. (2009). Acoustic Microscopy.

[3] Rupin F., Saïed A., Dalmas D., Peyrin F., Haupert S., Raum K., Barthel E., Boivin G., Laugier P. (2009). Assessment of Microelastic Properties of Bone Using Scanning Acoustic Microscopy: A Face-to-Face Comparison with Nanoindentation. Jpn. J. Appl. Phys..

[4] Lee Y.-C. (2001). Measurements of Multimode Leaky Lamb Waves Propagating in Metal Sheets. Jpn. J. Appl. Phys..

[5] Smolorz S., Grill W. (1996). Focusing PVDF transducers for acoustic microscopy. Res. Nondestruct. Eval..

[6] Merks E.J.W., Bouakaz A., Bom N., Lancee C.T., Van Der Steen A.F.W., De Jong N. (2006). Design of a multilayer transducer for acoustic bladder volume assessment. IEEE Trans. Ultrason. Ferroelectr. Freq. Control.

[7] Snook K., Rhee S., Robert M., Gottlieb E., Shung K.K. Development of P (VDF-TrFE) ultrasonic transducers operating at 50–120 MHz. Proceedings of the 2002 IEEE Ultrasonics Symposium.

[8] Lockwood G., Turnball D., Christopher D., Foster F. (1996). Beyond 30 MHz [applications of high-frequency ultrasound imaging]. IEEE Eng. Med. Boil. Mag..

[9] Ohigashi H., Koga K. (1982). Ferroelectric copolymers of vinylidenefluoride and trifluoroethylene with a large electromechanical coupling factor. Jpn. J. Appl. Phys..

[10] Zou W., Holland S., Kim K.Y., Sachse W. (2003). Wideband high-frequency line-focus PVDF transducer for materials characterization. Ultrason.

[11] Buckwalter J.A., Mankin H.J. (1998). Articular cartilage: Degeneration and osteoarthritis, repair, regeneration, and transplantation. Instr. Course Lect..

[12] Hagiwara Y., Saijo Y., Ando A., Onoda Y., Suda H., Chimoto E., Hatori K., Itoi E. (2012). Comparison of articular cartilage images assessed by high-frequency ultrasound microscope and scan-ning acoustic microscope. Int. Orthop..

[13] Nieminen H.J., Zheng Y.P., Saarakkala S., Wang Q., Toyras J., Huang Y.P., Jurvelin J.S. (2009). Quantitative assessment of articular cartilage using high-frequency ultrasound: Research findings and diagnostic prospects. Crit. Rev. Biomed. Eng..

[14] Myers S.L., Dines K., Brandt D.A., Brandt K.D., Albrecht M.E. (1995). Experimental assessment by high frequency ultrasound of articular cartilage thickness and osteoarthritic changes. J. Rheumatol..

[15] Männicke N., Schöne M., Oelze M., Raum K. (2014). Articular cartilage degeneration classification by means of high-frequency ultrasound. Osteoarthr. Cartil..

[16] Kuroki H., Nakagawa Y., Mori K., Kobayashi M., Yasura K., Okamoto Y., Suzuki T., Nishitani K., Nakamura T. (2008). Ultrasound properties of articular cartilage in the tibio-femoral joint in knee osteoarthritis: Relation to clini-cal assessment (International Cartilage Repair Society grade). Arthritis Res. Ther..

[17] Habib A., Melandsand F. Chirp coded ultrasonic pulses used for scanning acoustic microscopy. Proceedings of the 2017 IEEE International Ultrasonics Symposium (IUS).

[18] Aula A., Töyräs J., Tiitu V., Jurvelin J. (2010). Simultaneous ultrasound measurement of articular cartilage and subchondral bone. Osteoarthr. Cartil..

[19] Recht M., Bobic V., Burstein D., Disler D., Gold G., Gray M., Kramer J., Lang P., McCauley T., Winalski C. (2001). Magnetic resonance imaging of articular cartilage. Clin. Orthop. Relat. Res..

[20] Chu C.R., Lin D., Geisler J.L., Chu C.T., Fu F.H., Pan Y. (2004). Arthroscopic microscopy of articular cartilage using optical coherence tomography. Am. J. Sports Med..

[21] Mollenhauer J., Aurich M., Zhong Z., Muehleman C., Cole A., Hasnah M., Oltulu O., Kuettner K., Margulis A., Chapman L. (2002). Diffraction-enhanced X-ray imaging of articular cartilage. Osteoarthr. Cartil..

[22] Grassi W., Lamanna G., Farina A., Cervini C. (1999). Sonographic imaging of normal and osteoarthritic cartilage. Semin. Arthritis Rheum..

[23] Habib A., Shelke A., Vogel M., Pietsch U., Jiang X., Kundu T. (2012). Mechanical characterization of sintered piezo-electric ceramic material using scanning acoustic microscope. Ultrasonics.

[24] Habib A., Shelke A., Vogel M., Brand S., Jiang X., Pietsch U., Banerjee S., Kundu T. (2015). Quantitative Ultrasonic Characterization of c-Axis Oriented Polycrystalline AlN Thin Film for Smart Device Application. Acta Acust. United Acust..

[25] Hofmann M., Pflanzer R., Habib A., Shelke A., Bereiter-Hahn J., Bernd A., Kippenberger S. (2016). Scanning acoustic microscopy—A novel noninvasive method to determine tumor interstitial fluid pressure in a xenograft tumor model. Transl. Oncol..

[26] Laasanen M.S., Saarakkala S., Töyräs J., Hirvonen J., Rieppo J., Korhonen R.K., Jurvelin J.S. (2003). Ultrasound indentation of bovine knee articular cartilage in situ. J. Biomech..

[27] Saijo Y. (2009). Acoustic microscopy: Latest developments and applications. Imaging Med..

[28] Kim K., Wagner W.R. (2016). Non-invasive and non-destructive characterization of tissue engineered constructs using ultra-sound imaging technologies: A review. Ann. Biomed. Eng..

[29] Wagle S., Habib A., Melandsø F. (2017). Ultrasonic measurements of surface defects on flexible circuits using high-frequency focused polymer transducers. Jpn. J. Appl. Phys..

[30] Olympus High-Frequency Commercial Transducers. https://www.olympus-ims.com/en/ultrasonic-transducers/highfrequency/#!cms[focus]=cmsContent10879&cms[tab]=undefined.

[31] Habib A., Wagle S., Decharat A., Melandsø F. (2018). Numerical and Experimental Evaluation of High-Frequency Unfocused Polymer Transducer Arrays. Sensors.

[32] Habib A., Vierinen J., Islam A., Martinez I.Z., Melandso F. In Vitro Volume Imaging of Articular Cartilage Using Chirp-Coded High Frequency Ultrasound. Proceedings of the 2018 IEEE International Ultrasonics Symposium (IUS).

[33] Habib A., Wagle S., Decharat A., Melandsø F. (2020). Evaluation of adhesive-free focused high-frequency PVDF copolymer transducers fabricated on spherical cavities. Smart Mater. Struct..

[34] Younan Y., Aubry J.-F., Mickael T. (2014). Non-Invasive Therapy of Brain Disorders with Focused Ultrasound: From Animal Ex-Periments to Clinical Transfer. Ph.D. Thesis.

[35] Foster F., Harasiewicz K., Sherar M. (2000). A history of medical and biological imaging with polyvinylidene fluoride (PVDF) transducers. IEEE Trans. Ultrason. Ferroelectr. Freq. Control.

[36] Potter H.G., Black B.R., Chong L.R. (2009). New Techniques in Articular Cartilage Imaging. Clin. Sports Med..

